# CME ACTIVITY: National Outbreak of *Acanthamoeba* Keratitis Associated with Use of a Contact Lens Solution, United States

**DOI:** 10.3201/eid1508.0902251

**Published:** 2009-08

MedscapeCME is pleased to provide online continuing medical education (CME) for this journal article, allowing clinicians the opportunity to earn CME credit. This activity has been planned and implemented in accordance with the Essential Areas and policies of the Accreditation Council for Continuing Medical Education through the joint sponsorship of MedscapeCME and Emerging Infectious Diseases. MedscapeCME is accredited by the Accreditation Council for Continuing Medical Education (ACCME) to provide continuing medical education for physicians. MedscapeCME designates this educational activity for a maximum of 0.5 *AMA PRA Category 1 Credits*™. Physicians should only claim credit commensurate with the extent of their participation in the activity. All other clinicians completing this activity will be issued a certificate of participation. To participate in this journal CME activity: (1) review the learning objectives and author disclosures; (2) study the education content; (3) take the post-test and/or complete the evaluation at **http://www.medscape.com/cme/eid**; (4) view/print certificate.

## Learning Objectives

Upon completion of this activity, participants will be able to:

- Describe the incidence and etiology of *Acanthamoeba* keratitis (AK) infection in the United States
- Identify the demographic and clinical characteristics of patients who acquired AK
- Identify the risk factors for AK among contact lens users.

## EDITOR

**Carol Snarey**, Copyeditor, *Emerging Infectious Diseases*. *Disclosure: Carol Snarey has disclosed no relevant financial relationships.*

## CME AUTHOR

**Désirée Lie, MD, MSEd,** Clinical Professor, Family Medicine, University of California, Orange; Director, Division of Faculty Development, UCI Medical Center, Orange, California. *Disclosure: Désirée Lie, MD, MSEd, has disclosed no relevant financial relationships.*

## AUTHORS

*Disclosures:* ***Jennifer R. Verani, MD, MPH; Suchita A. Lorick, DO, MPH; Jonathan S. Yoder, MPH, MSW; Michael J. Beach, PhD; Christopher R. Braden, MD; Jacquelin M. Roberts, MS; Craig S. Conover, MD; Sue Chen, MPH; Kateesha A. McConnell, MPH; Douglas C. Chang, MD; Benjamin J. Park, MD; Dan B. Jones, MD; Govinda S. Visvesvara, PhD;*** and ***Sharon L. Roy, MD, MPH,*** *have disclosed no relevant financial relationships.*

## Earning CME Credit

To obtain credit, you should first read the journal article. After reading the article, you should be able to answer the following, related, multiple-choice questions. To complete the questions and earn continuing medical education (CME) credit, please go to **http://www.medscape.com/cme/eid**. Credit cannot be obtained for tests completed on paper, although you may use the worksheet below to keep a record of your answers. You must be a registered user on Medscape.com. If you are not registered on Medscape.com, please click on the New Users: Free Registration link on the left hand side of the website to register. Only one answer is correct for each question. Once you successfully answer all post-test questions you will be able to view and/or print your certificate. For questions regarding the content of this activity, contact the accredited provider, CME@medscape.net. For technical assistance, contact CME@webmd.net. American Medical Association’s Physician’s Recognition Award (AMA PRA) credits are accepted in the US as evidence of participation in CME activities. For further information on this award, please refer to http://www.ama-assn.org/ama/pub/category/2922.html. The AMA has determined that physicians not licensed in the US who participate in this CME activity are eligible for *AMA PRA Category 1 Credits*™. Through agreements that the AMA has made with agencies in some countries, AMA PRA credit is acceptable as evidence of participation in CME activities. If you are not licensed in the US and want to obtain an AMA PRA CME credit, please complete the questions online, print the certificate and present it to your national medical association.

### Article Title: National Outbreak of *Acanthamoeba* Keratitis Associated with Use of Contact Lens Solution, United States

## CME Questions

1. Which of the following most accurately describes the incidence and characteristics of Acanthamoeba keratitis (AK) in the United States?
  A. Occurs primarily among hard contact lens users
  B. Annual incidence is 1–2 cases per 1 million
  C. A painless corneal infection
  D. Has a benign course
2. Which of the following is least likely to describe the characteristics of AK associated with the cases reported in this article?
  A. Median patient age was 40 years
  B. Most used a contact lens cleaning solution
  C. Most used soft contact lenses
  D. Median time to treatment was 49 days
3. Which of the following is most likely to be an independent predictor of AK in contact lens users?
  A. Swimming in lakes while wearing lenses
  B. Hispanic ethnicity
  C. Topping off lens solutions
  D. Lack of handwashing before lens insertion

### Activity Evaluation

**Table:** 

**1. The activity supported the learning objectives.**				
Strongly Disagree				Strongly Agree
1	2	3	4	5
**2. The material was organized clearly for learning to occur.**				
Strongly Disagree				Strongly Agree
1	2	3	4	5
**3. The content learned from this activity will impact my practice.**				
Strongly Disagree				Strongly Agree
1	2	3	4	5
**4. The activity was presented objectively and free of commercial bias.**				
Strongly Disagree				Strongly Agree
1	2	3	4	5

